# Supplementary material for: TMC1 is an essential component of a leak channel that modulates tonotopy and excitability of auditory hair cells in mice
Source: eLife. 2019 Oct 29;8:e47441. doi: 10.7554/eLife.47441 (PMC6853638; doi:10.7554/eLife.47441)
Supplement: Supplementary file 1. — Specific primers were designed for PCR of the Tmc1-deafness vector and amino-acid-substituted Tmc1 constructs, based on the pCDNA3.1 vector containing mouse Tmc1 cDNA. DF, deafness; F, forward; R, reverse. [file elife-47441-supp1.docx]

| Primers | 5'-3' |
| --- | --- |
| TMC1-DF-F | tgagattaacaacaaggaattcgtgcgtctcaccgttt |
| TMC1-DF-R | tgagacgcacgaattccttgttgttaatctcatccatcaaggc |
| mTMC1-G411C-F | aatgtccctcctgTGTatgttctgtcccaccctgtttga |
| mTMC1-G411C-R | ACAcaggagggacattaccatgttcatttcatttttttcccacca |
| mTMC1-M412C-F | gtccctcctggggTGTttctgtcccaccctgtttgactt |
| mTMC1-M412C-R | ACAccccaggagggacattaccatgttcatttcatttttttccca |
| mTMC1-N447C-F | tcttcttctaggcTGTttgtatgtattcattctcgcctt |
| mTMC1-N447C-R | ACAgcctagaagaagagcaaaaatgcgccccaggag |
| mTMC1-D528C-F | tctcaccgtttctTGTgtcctgaccacttacgtcacgat |
| mTMC1-D528C-R | ACAagaaacggtgagacgcacgaattcctgccccaccattgtttc |
| mTMC1-T532C-F | tgacgtcctgaccTGTtacgtcacgatcctcattggcga |
| mTMC1-T532C-R | ACAggtcaggacgtcagaaacggtgagacgcacgaattc |
| mTMC1-D569C-F | atacacagaattcTGTatcagtggcaacgtcctcgctct |
| mTMC1-D569C-R | ACAgaattctgtgtatgaaggatatccatattctaagtcccagca |

**Supplementary File 1.** Primers used for generating desired truncation and mutations in mouse *Tmc1* cDNA.
