# Supplementary material for: TMC1 is an essential component of a leak channel that modulates tonotopy and excitability of auditory hair cells in mice
Source: eLife. 2019 Oct 29;8:e47441. doi: 10.7554/eLife.47441 (PMC6853638; doi:10.7554/eLife.47441)
Supplement: Transparent reporting form [file elife-47441-transrepform.pdf]

eLIFE
1st Floor
24 Hills Road
Cambridge CB2 1JP, UK
P 01223 855340
W elifesciences.org
T @elife

eLife's transparent reporting form

We encourage authors to provide detailed information *within their submission* to facilitate the interpretation and replication of experiments. Authors can upload supporting documentation to indicate the use of appropriate reporting guidelines for health-related research (see EQUATOR Network), life science research (see the BioSharing Information Resource), or the ARRIVE guidelines for reporting work involving animal research. Where applicable, authors should refer to any relevant reporting standards documents in this form.

If you have any questions, please consult our Journal Policies and/or contact us: editorial@elifesciences.org.

Sample-size estimation

- You should state whether an appropriate sample size was computed when the study was being designed
- You should state the statistical method of sample size computation and any required assumptions
- If no explicit power analysis was used, you should describe how you decided what sample (replicate) size (number) to use

Please outline where this information can be found within the submission (e.g., sections or figure legends), or explain why this information doesn't apply to your submission:

As described from line 681 to line 684: Every experiments contained at least 3 biological replicates and over 10 cell numbers. For certain experiment such as single channel recording, the traces number were over 100. All cell numbers were noted in the figure legends.

Replicates

- You should report how often each experiment was performed
- You should include a definition of biological versus technical replication
- The data obtained should be provided and sufficient information should be provided to indicate the number of independent biological and/or technical replicates
- If you encountered any outliers, you should describe how these were handled
- Criteria for exclusion/inclusion of data should be clearly stated
- High-throughput sequence data should be uploaded before submission, with a private link for reviewers provided (these are available from both GEO and ArrayExpress)

Please outline where this information can be found within the submission (e.g., sections or figure legends), or explain why this information doesn't apply to your submission:

1. For most of experiments, the data were collected at least every 2 weeks within 3 months for steady condition. (line 681-682)

2. Multiple recordings from one cell with the identical stimulus protocol were considered as technical replications, which were averaged to generate a single biological replication representing value/data from one cell. (line 684-686)

3. N number in Fig.1 to Fig.8 represent for cells recorded, in Fig.9B, the n/N number means events/cells numbers. All this information was described in corresponding figure legend.

4. We did not encounter any outliers.

5. For single channel recording data, only traces with obvious single channel events have been included for analyzing. (line 622-623)

6. No high-throughput sequence data have been used.

Statistical reporting

- Statistical analysis methods should be described and justified
- Raw data should be presented in figures whenever informative to do so (typically when N per group is less than 10)
- For each experiment, you should identify the statistical tests used, exact values of N, definitions of center, methods of multiple test correction, and dispersion and precision measures (e.g., mean, median, SD, SEM, confidence intervals; and, for the major substantive results, a measure of effect size (e.g., Pearson's r, Cohen's d)
- Report exact p-values wherever possible alongside the summary statistics and 95% confidence intervals. These should be reported for all key questions and not only when the p-value is less than 0.05.

Please outline where this information can be found within the submission (e.g., sections or figure legends), or explain why this information doesn't apply to your submission:

1. Statistical analysis methods was described in Materials and Methods (line 680 to 689).

2. In each figure, the raw data were showed as dots within each bar chart (Fig.1 C D, Fig.2 D F,Fig.3 C D, Fig.4 D, Fig.6 A B D, Fig.7 B C D G H, Fig.8 C E) . Because of the requirement for plotting, panels with curve fitting did not show every raw data point (Fig.1 F G, Fig.5 B C D E H, Fig.6 F G, Fig.7 I, Fig.9 B). Mostly, N number were more than 10.

3. N number, mean, SD/SEM, and etc. were described in each figure legend and labeled on corresponding panels (Fig.1 to Fig.9, SFig.2).

4. p-values has been described in each figure legend and labeled on corresponding panels (Fig.1 to Fig.9, SFig.2).

(For large datasets, or papers with a very large number of statistical tests, you may upload a single table file with tests, Ns, etc., with reference to sections in the manuscript.)

Group allocation

- Indicate how samples were allocated into experimental groups (in the case of clinical studies, please specify allocation to treatment method); if randomization was used, please also state if restricted randomization was applied
- Indicate if masking was used during group allocation, data collection and/or data analysis

Please outline where this information can be found within the submission (e.g., sections or figure legends), or explain why this information doesn't apply to your submission:

eLife Sciences Publications, Ltd is a limited liability non-profit non-stock corporation incorporated in the State of Delaware, USA, with company number 5030732, and is registered in the UK with company number FC030576 and branch number BR015634 at the address 1st Floor, 24 Hills Road, Cambridge CB2 1JP | August 2014

For *ex vivo* tissue recording, experimental groups and control groups were allocated according to the genotype of animals. Description of animal sources was in lines from 575 to 579. For injectoporation experiments, experimental groups and control groups were allocated according to the injected cDNA plasmids, as described in sentence from line 581 to line 591.

Additional data files ("source data")

- We encourage you to upload relevant additional data files, such as numerical data that are represented as a graph in a figure, or as a summary table
- Where provided, these should be in the most useful format, and they can be uploaded as "Source data" files linked to a main figure or table
- Include model definition files including the full list of parameters used
- Include code used for data analysis (e.g., R, MatLab)
- Avoid stating that data files are "available upon request"

Please indicate the figures or tables for which source data files have been provided:

N/A
